# Sex Offenses Perpetrated Against Older Adults: A Multivariate Analysis of Crime Scene Behaviors

**DOI:** 10.1177/0886260520928639

**Published:** 2020-06-15

**Authors:** Louise Almond, Martha Sainsbury, Michelle McManus

**Affiliations:** 1 University of Liverpool, UK; 2 Liverpool John Moores University, UK

**Keywords:** sexual assault, elder abuse, anything related to sexual assault

## Abstract

The aim of this study was to thematically explore the relationship between crime scene behaviors and background characteristics of offenders who commit sexual offenses against female victims aged 60 years or more. Research and understanding of offense behaviors in this area is extremely limited; therefore, the study sought to provide a preliminary understanding and multivariate model of offense behaviors in cases where older female adults were sexually abused. Twenty-seven crime scene behaviors from 143 rape or attempted rape cases of an older adult victim were analyzed; frequency data were computed to provide base rate information, and Smallest Space Analysis provided a visual representation of the co-occurrence of crime scene behaviors. Three distinct dominant themes were identified, in which 56% of offenses displayed themes of *Involvement* (22%), *Control* (17%), and *Hostility* (16%). The relationship between each dominant theme and selected background characteristics was then analyzed. For example, offenders displaying an *Involvement* theme were found to be significantly less likely to have prior convictions. Significance was also found in the relationship between dominant themes and a “theft and kindred offence other” preconviction background characteristic. The findings demonstrate offending behavior can be separated into three distinct themes, providing an explanation of offender subtypes and supporting previous models found in other types of sexual offending. Applications for law enforcement agencies regarding identified themes and links with likely offender background characteristics are highlighted. Limitations and future research avenues are discussed.

## Introduction

Sexual offending is a pervasive crime within society and has received considerable academic research in efforts to understand the crime, the type of offender involved, and its impact on victims. For example, recent statistics indicate 3.9% of adults (648,000 victims) aged 16 through 59 years had been victims of a sexual offense within the United Kingdom in the previous year (Office for National Statistics [ONS], 2018). However, sexual assaults against an older adult victim has been largely absent from much of the sexual offending research. The Crime Survey for England and Wales only increased the respondent age range for its primary self-completion survey from those aged 16 through 59 to those aged 16 through 74 years in April 2017 (ONS, 2018). Therefore, reports prior to April 2017 did not include any victims older than 59 years. This further highlights the potential lack of national understanding around older adult individuals’ experiences with domestic and sexual violence.

Current research provides a limited understanding of this crime. Estimates indicate older adult victims comprise between 0.2% and 5.2% of all penetration rape and sexual assaults reported to the police (Bows, 2018). Mirroring the pattern of sexual offending in general, the majority of older victims are female, and perpetrators are overwhelmingly male (Bows & Westmarland, 2015). Offenders tend to commit offenses individually (Ball & Fowler, 2008), and victims are likely to know the perpetrator (Bows & Westmarland, 2015).

Individuals offending against older adult victims are significantly more likely to have prior convictions than those who assault younger victims (Lea et al., 2011). Burglary is the most prominent prior conviction (Safarik et al., 2002); however, prior sexual convictions were found among one third of this perpetrator cohort (Jeary, 2005).

The typical age of offenders has been debated among researchers, some proposing a higher mean age of 34.67 years (Ball & Fowler, 2008), others finding the majority of offenders under age 30 years (Jeary, 2005), or display no significant difference when compared with the age of those offending against younger victims (Lea et al., 2011). Others argue offenders are in fact heterogeneous in nature, displaying noteworthy variance (Burgess et al., 2007), indicating a need for further research to develop a better understanding.

Much work has been done on chronophilia, understood as age-based sexual attractions (Seto, 2016). Atypical chronophilia have been defined as follows: nepiophilia (infant/toddlers), pedophilia (prepubescent children), hebephilia (pubescent children), ephebophilia (postpubescent, sexually maturing adolescents), teleiophilia (young sexually mature adults, typically 20 s and 30 s), mesophilia (middle-aged adults, typically 40 s and 50 s), and gerontophilia (older adult adults, typically 60 s and older) (Seto, 2016). However, very little work has been published on gerontophilia, with research often singling out individual case studies (Kaul & Duffy, 1991). As a result, Ball (2005) describes gerontophilia as a

poorly described, defined and understood preference that may also be a paraphilia and compulsion characterized by a sexual preference for older partners. There is no agreed definition; prevalence and aetiology are unknown as systematic studies have not been undertaken. (p. 1)

Most studies in the limited field of sexual offending against older adults have focused on the offender, leaving crime scene behaviors and offense information significantly underresearched. Lea et al. (2011) found that a surprise initial approach was more often preferred by offenders against older as opposed to younger adult victims, and discussion among researchers has focused on the level of violence inflicted during assaults against older victims. For example, some researchers suggest older adult victims do not experience more violence than younger victims (Ball & Fowler, 2008), whereas others argue violence frequently exceeds the levels necessary for compliance (Jeary, 2005). Safarik et al. (2002) found older adults were less likely to fight back and more likely to sustain injuries during an offense. This suggests that victim vulnerability rather than excessive force may offer an explanation for perceived levels of violence.

Causation theories for gerontophilia within the limited research suggest a “displacement of incestuous wishes” and/or “early sexual experience resulting in erotic-sexual fixation of age disparity” (Ball, 2005, p. 1). However, all suggested explanations and hypothetical assumptions in this field should be considered with caution due to the finite literature base available.

Published literature investigating the impact of sexual offending on older victims is similarly restricted, highlighting the importance of and need for further research. Older adult victims have experienced bone fractures (Stöckl & Penhale, 2015), musculoskeletal trauma (Disney & Cupitt, 2000), increased risk of heart disease and stroke (Smith & Breiding, 2011), and overall poor mental health (Fisher & Regan, 2006; Soares et al., 2010). Burgess et al. (2000) found 11 out of 20 older adult victims sampled died within or around a year following their attacks, an extreme outcome clearly demonstrating the potential value of further research. The importance of further research is further highlighted by World Health Organization (WHO) statistics indicating the population of individuals over the age of 60 years, potential victims of elder sexual abuse, will more than double to approximately 2 billion by 2050 (WHO, 2018).

A significant weakness in the current body of literature is the lack of a collective definition of an older adult victim. The WHO (2018) defines an older adult person to be any individual over the age of 60 years, while stressing that their definition may vary depending on culture and living conditions. For example, in Western societies, the chronological age of 65 years may be accepted as defining an older adult, whereas in parts of the developing world, the definition may be age of 50 or 55 years.

The literature displays similar variability ranging from victims age 50 (Bows, 2018) to 66 years (O’Keeffe et al., 2007) making generalizability across the literature difficult. In the United Kingdom, national sex offending census data of person older than 59 years was not collected prior to 2017 (ONS, 2018). For present purposes, the definition of an older adult victim will be stipulated as being any person 60 years or more.

Census data concerning older adult victims could be invaluable to future research and may have been so to this study. This is because, as Bows and Westmarland (2015) propose, underreporting to police may be particularly high in older adult victims because offenses against them lack similarity or “fit” to the typical “real-rape” stereotype which relates to the myth of rape being an outdoors attack on a young woman. Older adult victims present obvious age differences and have a significantly higher likelihood of being attacked in their own home (Lea et al., 2011); victims, therefore, may believe they will have to convince police of their account (McMillan & Thomas, 2009). In addition, society tends to perceive older adult individuals as asexual (Montemurro & Siefken, 2014), thus decreasing the likelihood of older adult individuals would be viewed as potential rape victims. Lea et al. (2011) builds on this by suggesting that friends and family of older adult victims may fail to identify signs of attack due to their own preconceptions of the typical victim, further preventing disclosure and reporting.

Therefore, it is clear that the current body of available research lacks a detailed understanding of sexual offending against the older adult and an attempt at identifying themes within offending behavior has yet to be made in respect of this specific victim group. Because of this, past researchers have drawn upon general sexual offending literature, in which multivariate models have been created, to suggest likely themes. Although past studies each investigated different subsamples of sex offender, high levels of commonality have been found for the three themes, *control*, *hostility*, and *involvement*, each of which was considered in this study.

### Control

Previous literature identifying “Control” as a dominant theme suggests offenders use their victim simply as a tool for their own sexual and instrumental gratification, hence behaviors reflecting lack of empathy and high control are common (Almond & Canter, 2007; Canter & Youngs, 2012). These include, for example, stealing from the victim or forcing participation in sexual acts (Canter, 1994). Offenses displaying this theme tend to be opportunistic with the offender paying little attention to the features of the victim because they are perceived as an object, rather than a person (Almond et al., 2014). Vulnerable individuals are likely to be victim to this type of offense (Salfati & Canter, 1999).

### Hostility

The dominant theme of “Hostility” is characterized by anger and violent behavior resulting in victims experiencing levels of emotional and physical aggression beyond that needed for compliance (Canter et al., 1998, 2003). Many researchers have proposed that this theme involves offenders viewing the victim as a vehicle to vent frustration (Almond, McManus, Giles, & Houston, 2017). A hostile dominant theme has been identified in many studies of sexual offending including internet facilitated rape (Almond, McManus, & Chatterton, 2017) and male-on-male sexual assault (Almond et al., 2014). Hence, it is reasonable to assume a *hostility* dominant theme was also likely to be identified in the present work.

### Involvement

Perpetrators demonstrating “Involvement” tend to be using their offending behavior to seek intimacy (Marshall, 1989), or to compensate for a lack of social abilities (Seidman et al., 1994). Research indicates offenders create a pseudo-intimate relationship and, in contrast to *Hostile* offenders, treat the victim as a person rather than an object (Canter, 1994). Thus, behaviors shown reflect the offender’s belief that the victim is a reactive individual. Offenders are likely to talk to victims, particularly complimenting, or reassuring them (Almond et al., 2014). The sexual behaviors chosen are likely to demonstrate parallels to normal sexual behavior such as kissing and reciprocal sexual acts, suggesting individuals are compensating for a lack of social contact (Almond et al., 2014). An *Involvement* theme is regularly found in previous literature (Almond et al., 2014; Almond, McManus, Giles, & Houston, 2017), supporting the assumption that a similar theme was likely to be identified in the present study.

### Aims of the Present Study

Given the paucity of age-specific research literature identified above, and having established a sample suitable for the purpose, the first aim of this study is to provide significant base rate information concerning crime scene behaviors and characteristics of both offenders and victims. The research then aimed to explore offender crime scene behaviors for dominant themes before finally investigating potential associations revealed by the study between offender characteristics and any dominant behavioral themes uncovered.

## Method

### Sample

The study sample consisted of 143 rape and attempted rape cases reported to the police between 2003 and 2017 involving a female victim aged 60 years and over in the Violent Crime Linkage Analysis System (ViCLAS) database. Of these, 68 of the cases had led to convictions so that offender characteristics were known.

Data were only provided for single offender cases, and the final sample selected only included female victims. Cases involving male victims were extremely rare and including them would impact on the output representative of female victims, while providing nonrepresentative findings for male victims and creating problems for generalization as a whole.

### Variables

Precoded data were obtained from the ViCLAS database, through a partnership with the National Crime Agency and SCAS (Serious Crime Analysis Section). Twenty-seven crime scene behavior variables were extracted associated with offending behavior such as weapon used, disrobement, and sex acts. For each case, behaviors were precoded as either present or absent by SCAS. It is usual practice to include all cases where variables occur at a higher frequency than 5% (Almond et al., 2014; Almond, McManus, & Chatterton, 2017); however, 67 variables were extracted using this method, which was considered too large a sample to adequately identify dominant themes. Variables occurring at a frequency of less than 10% of cases were, therefore, excluded from inferential analysis because such low prevalence provides little information, yet may impact significant findings (Goodwill & Alison, 2007). Similarly, crime scene behaviors occurring in 70% or more cases were removed from the inferential analysis because behaviors occurring in such a clear majority of cases provide little information when differentiating between cases and identifying themes (Almond & Canter, 2007).

### Statistical Analysis

Frequency data were analyzed to gain a preliminary understanding of offending behavior. Smallest Space Analysis (SSA) was used to provide a 2D visual geometric diagram representing the relationship of each variable with all others within the analysis. Each crime scene behavior is represented by a point; the distance between points represents the relationship of each behavior with all other behaviors within the analysis. Variables occurring frequently together are closer together within the space (Guttman, 1968). This multidimensional scaling procedure is used to highlight links and patterns between variables, particularly subtle connections which may not be identified through other analysis techniques. Due to the possibility of behaviors not being recorded when in fact they did occur within police data, Jaccard’s coefficient was used as it benefits from the ability to ignore joint nonoccurrence’s (Canter et al., 2003). Following interpretation of the SSA output, each case was then explored individually for any dominant theme of behavior disclosed. Next, Chi-square analyses were computed to map offender and victim background characteristics onto those offenders who presented with a dominant theme to test their level of association. Finally, Kruskal–Wallis test was used in the same way for continuous variables such as age.

### Preliminary Analysis

Preliminary age analysis was undertaken to ensure that significant differentiation in crime scene behaviors did not occur across age groups within the sample. This was important because the available literature has shown significant variation in the definition of an “older adult” victim. Age groups were separated into two, victims aged from 60 to 69 years (*n = *65) and 70+ years (*n = *78). Findings indicated only three out of the 27 crime scene behaviors displayed significant differences between age groups, these being Self-Disclosure, χ^2^(1) = 8.726, *p* = .003, Victim Naked, χ^2^(1) = 7.246, *p* = .007, and Victim Reporting, χ^2^(1) = 5.091, *p* = .024. All three behaviors were more likely to occur in attacks involving younger victims (60–69); however, as the findings overall indicated because there were so few significant differences between age groups, this provided a clear rationale to treat the entire sample as a cohesive group.

## Results

### Base Rate Statistics

Frequency analysis of all information provided from the ViCLAS database was computed to produce base rate information.

#### Victim characteristics (*N* = 143)

The average age of victim was 72.99 (*SD* = 9.99) with ages ranging from 60 to 101 years. Victims of offenses on the ViCLAS database were overwhelmingly White European (96%, *n* = 137), with a small minority being African Caribbean (1%, *n* = 1) and Oriental (1%, *n* = 1). In 85% (*n* = 122) of offenses, the offender was a stranger to the victim, 7% (*n* = 10) had some peripheral contact prior to the offense, and in 4% (*n* = 5) of offenses, the victim and offender were known friends.

#### Offender characteristics (*n* = 68)

Seventy-one percent of the 68 convicted offenders identified as White European, 13% (*n* = 9) African Caribbean, and 7% (*n* = 5) Asian. The average age of convicted offenders at the time of the offense (*n* = 68) was 31.74 (*SD* = 12.34) (range = 14–63) years. Where gender was known, all offenders were male.

Of the 68 offenders who were convicted of sexual offending against individuals aged 60 years or above, 74% (*n* = 50) had been convicted of previous offenses, totaling 890 prior convictions. See Table 1

**Table 1.** Preconviction Frequency Results (*n* = 68).

Preconviction (*n* = 68)	Frequency of Offenders *n* (%)
Theft	31 (46)
Offenses related to police/prison/court	26 (38)
Criminal damage	25 (37)
Other theft and kindred	25 (37)
Burglary	24 (35)
Public disorder and rioting	17 (25)
Common assault	14 (21)
Minor traffic offenses	13 (19)
Offensive weapon/firearm	13 (19)
Other miscellaneous	12 (18)
Serious assault	11 (16)
Drug	10 (15)
Robbery	10 (15)
Fraud and kindred offenses	9 (13)
Harassment	7 (10)
Rape	7 (10)
Indecent assault	6 (9)
Sex offender registration	4 (6)
Going equipped	3 (4)
Indecent exposure	2 (3)
Manslaughter	2 (3)
Murder	2 (3)
Unlawful sexual intercourse	2 (3)
Other offenses against the person	2 (3)
Aggravated burglary	1 (1)
Buggery and gross indecency	1 (1)

for frequency and percentages of offender with preconvictions.

#### Offense

The majority of offenses took place indoors (79%, *n* = 113), while a minority occurred outdoors (22%, *n* = 31). Similarly, offenses were likely to occur in darkness (64%, *n* = 92) rather than in daylight (35%, *n* = 51). It was very common for offenses to take place at the victim residence (74%, *n* = 106), with a small number occurring at the offender residence (3%, *n* = 4) or a nursing home (2%, *n* = 3). When the offense was recorded as outdoors, alleyways (4%, *n* = 6), forests (4%, *n* = 6), and streets (4%, *n* = 6) were the offense locations with the highest frequency.

#### Crime scene behaviors

In terms of initial approach, surprise (55%, *n* = 79) and confidence (45%, *n* = 64) were frequently used. Among surprise approaches, snuck upon (31%, *n* = 44) and while the victim slept (20%, *n* = 29) were the most common tactics. Among confidence approaches, the most common tactic was engaged in conversation with the victim (15%, *n* = 21). In 29% (*n* = 41) of cases, property was stolen.

Violence occurred during 21% (*n* = 30) of the cases, 17% (*n* = 24) after the victim resisted, and 15% (*n* = 21) after contact, in only 2% (*n* = 3) of offenses did violence continue after the incident. Perpetrators were most likely to show a minimal level of force (24%, *n* = 34); however, moderate force (14%, *n* = 20) and severe force (3%, *n* = 4) also occurred. In 11% (*n* = 16) of the assaults, no injury was reported. “Extreme overkill” as a level of force was not present in any of the offenses in this sample. Cases where this level of force is used are most likely to have been recorded as an attempted murder and, therefore, did not meet this study’s offense search criteria. Within the sample, victims presented with various injury locations, the most common were to the face (22%, *n* = 31), neck (13%, *n* = 19), and head (8%, *n* = 11).

Some level of disrobement was frequently recorded, most commonly shown by the victims clothing moved to expose (36%, *n* = 51), or partially disrobed, and were clothing is removed (30%, *n* = 43). However, both victim (18%, *n* = 26) and offender (11%, *n* = 16) were naked in a frequent number of offenses. The reason why so many victims were naked may be because they were sleeping at the time of the initial contact (20%, *n* = 29). Offenders tended to disrobe themselves (74%, *n* = 106) and their victims (64%, *n* = 92), yet in a small number of cases victims disrobed themselves (17%, *n* = 24).

#### Inferential analysis

**Figure 1.**
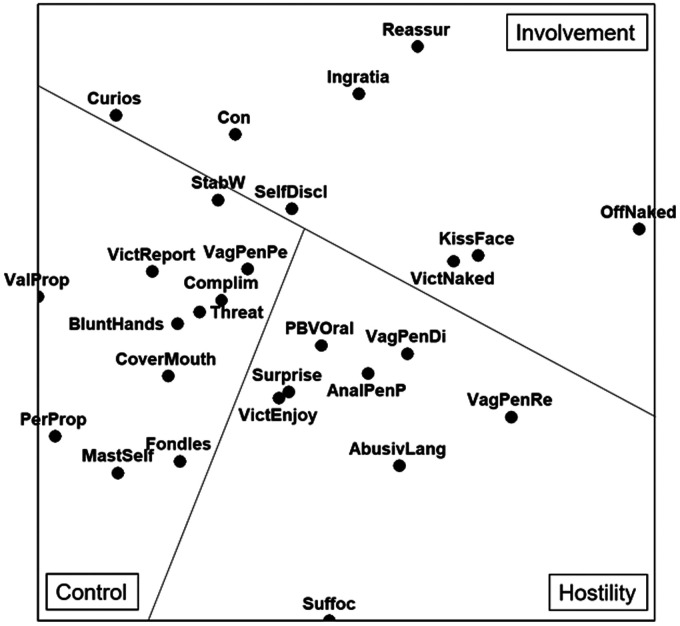
Smallest Space Analysis of crime scene actions and behavior themes of sex offences committed against older adults.

An SSA was computed on 27 crime scene offenses across 143 cases. The output produced a coefficient of alienation of .21 indicating an acceptable practical “fit” between the current study and the original association matrix (Canter & Heritage, 1990; Shye et al., 1994). Figure 1

displays Vectors 1 and 3 of the three-dimensional space created by the SSA. Explanations and frequency results for crime scene behaviors are provided in Table 2

**Table 2.** Behavioral Composition of Themes.

Theme	Name	Explanation	*n* (%)
Control	VagPenPe	Vaginal penetration (penile)	82 (57)
	Threat	Offender verbally threatens victim	51 (36)
	Fondles	Offender fondles victim	37 (26)
	BluntHands	Blunt hand force used	37 (26)
	ValProp	Valuable property stolen	33 (23)
	CoverMouth	Offender covers victims mouth	32 (22)
	VictReport	Offender refers to victim reporting offense	26 (18)
	StabW	Stabbing Weapon used or displayed	24 (17)
	PerProp	Personal property stolen	22 (15)
	MastSelf	Offender masturbates self	19 (13)
	Complim	Offender compliments victim	19 (13)
Hostility	Surprise	Surprise initial approach used	78 (55)
	VagPenDi	Vaginal penetration (digit)	34 (24)
	PBVOral	Oral sex performed by victim	29 (20)
	AnalPenP	Anal penetration (penile)	29 (20)
	VagPenRe	Vaginal penetration (rear)	20 (14)
	VictEnjoy	Offender refers to victim enjoyment	17 (12)
	AbusivLang	Offender uses abusive language	17 (12)
	Suffoc	Offender manually suffocates victim	15 (11)
Involvement	Con	Con initial approach used	64 (45)
	SelfDiscl	Offender discloses information about themselves	58 (41)
	KissFace	Offender kisses victim’s face	27 (19)
	VictNaked	Victim naked	26 (18)
	Curios	Offender displays curiosity	24 (17)
	Reassur	Offender reassures victim	21 (15)
	Ingratia	Offender displays ingratiating behavior	16 (11)
	OffNaked	Offender naked	16 (11)

. The SSA output was examined and three distinct themes were successfully identified using a similar method to Almond, McManus, and Chatterton (2017).

##### Control

Variables in the left of Figure 1 demonstrate the offenders need for control within the offense. “Vaginal penetration (penile)” (57%) and “offender verbally threatens victim” (36%) displayed the highest frequency within the allocated theme. This supports the argument that offenders tend to exhibit controlling behaviors to achieve their sexual aims (Canter, 1994). The inclusion of variables such as “offender verbally threatens victim” and “offender refers to victim reporting offence” further supports the offenders need for control, demonstrating parallels to Canters (1994) “victim as object” theme.

Variables such as “vaginal penetration (penile),” “offender fondles victim,” and “offender masturbates self” support the sexual element regularly found within this theme (Almond et al., 2014; Almond, McManus, Giles, & Houston, 2017). This further supports the theory that sexual gratification is central to *Control* within the offender’s motivation. In addition, the inclusion of both “valuable property stolen” and “personal property stolen” indicates that instrumental gratification may also be desired by these offenders (Almond et al., 2014). Stealing items of sentimental value may further the offender’s feelings of control. Similarly, the precautionary behavior of “offender covers victims’ mouth” may display a level of criminal sophistication but is also another way to display control over the victim. Likewise, the use or display of a “stabbing weapon” demonstrates both an intentional desire to control and a level of preparedness. Parallels can be identified between these actions and Canter’s (1994) “Victim as object” and Almond, McManus, and Chatterton’s (2017) “Criminal sophistication” theme.

Unexpectedly and unusually “offender compliments victim” was also found in the “Control” section, a variable which tends to be included in *Involvement* themes elsewhere in the literature. This could be a way of the offender controlling the victim through “positive” behaviors; however, further research would be needed to fully understand why offenders displaying a high number of controlling behaviors are also likely to compliment the victim.

##### Hostility

Behaviors in the bottom right of Figure 1 have been identified as hostile. A “surprise” initial approach (55%) was the highest in frequency, indicating a hostile beginning to the offense. Although usually characterized by an abrupt start and immediate force (Canter & Heritage, 1990), it also includes circumstances in which the victim is sleeping and is “surprised” by the offender. The presence of “offender manually suffocates victim” and “offender uses abusive language” affirms the apparent violence demonstrated by offenders through both physical and verbal means. The identification of violent behaviors is consistent with previous literature and reflects overt aggression toward the victim.

The theme arguably demonstrates a highly sexualized motive with the inclusion of “vaginal penetration (digit),” “vaginal penetration (rear),” and “anal penetration (penile).” The presence here of “oral sex performed by victim” suggests the victim is forced to play an active role, and is an unexpected finding because previous research has found minimal sexual behaviors present within the *Hostility* theme (Almond et al., 2014). However, the inclusion of sexual behaviors has been argued to be an action to degrade, or further humiliate the victim, rather than achieve sexual gratification (Almond et al., 2015). This is supported by the type of sexual behaviors demonstrated in the theme, particularly “oral sex performed by victim,” “vaginal penetration (digit),” and “anal penetration (penile)” which could be identified as humiliating sexual behaviors within in a nonconsenting setting. In addition, the inclusion of sexual behaviors with hostile violence is comparable with Canter’s (1994) “victim as vehicle” theme.

Similarly, the presence of “offender refers to victim enjoyment” is also inconsistent with previous findings, it being more usually identified with an *Involvement* theme (Almond et al., 2014). It could be argued to be another form of sexual violence, particularly if the offender is suggesting the victim is enjoying the incident; or perhaps an act of intimidation, similar to the presence of “offender implies knowledge of victim” within the “*violence*” theme identified by Almond, McManus, and Chatterton (2017) in “internet Facilitated Rape.” Whatever the explanation, the increased presence of sexual behaviors in the *hostility* theme revealed in this study further highlights the particular vulnerability of this cohort of older adult victims. As shown from previous research and from the current sample (74%), older adult sexual offenses were more likely to occur in the home of the victim (Lea et al., 2011), or in care homes, which may allow an opportunity to inflict sustained, more hostile and violent sexual behaviors.

##### Involvement

In Figure 1, behaviors heading the analysis output reflect an *Involvement* theme characterized by the offender’s attempt to establish an interpersonal relationship with the victim. “Con approach” (45%) and “self-disclosure” (41%) were the highest frequency variables here. The use of a “con” initial approach could demonstrate the offenders attempt to create a “fake” relationship between victim and offender, showing parallels to the grooming behaviors found in Almond, McManus, Giles, and Houston’s (2017) “Involvement theme for female sex offenders.” Equally, it may be argued that a more vulnerable victim cohort may require some form of prior “con” engagement to facilitate access, especially should the preferred locus of offense be in the victim’s home or care home as discussed above.

Verbal behaviors such as “offender displays curiosity,” “offender reassures victim,” or “offender displays ingratiating behavior” also point to an offender’s attempt at an intimate relationship. Moreover, sexual behaviors such as “kisses face”; “offender naked,” or “victim naked” suggest similarities to expected conventions within a consensual sexual relationship. The small numbers of sexual behaviors and high levels of interaction with the victim within this theme display a “likeness to the victim as person” theme identified by Almond and Canter (2007) and Canter (1994). However, these behaviors could also reflect both increased time the offender may have with an older adult victim given the likely residential attack location with minimal risk of interruption, and the victim’s vulnerability meaning they are less likely to try and escape or fight the offender.

#### Dominant behavioral theme

To examine the ability of the framework to characterize individual offenses, each of the 143 cases was examined independently to identify a potential dominant theme. For each case, a score was allocated to each theme dependent on the percentage of relevant behaviors shown by the offender. To assign a dominant theme, the percentage score for one theme had to be greater or equal to the combined percentage score of the other two themes (Almond, McManus, & Chatterton, 2017). Hybrid cases were created if the above method could not isolate a single dominant theme and two themes displayed similarly high percentage scores. Those cases displaying neither dominant nor hybrid characteristics were deemed unclassifiable.

Using this method, 56% of the cases were assigned a dominant theme and 23% were classified as hybrids. Of those allocated a dominant theme, 22% were described as “Involvement,” 16% as “Hostile,” and 17% were classified as “Control” offenses. Of those allocated to hybrid themes, “Hostility/Control” was the most prevalent (11%), followed by “Involvement/Control” (10%) and “Involvement/Hostility” (3%). Twenty-one percent of cases displayed no clear theme and were therefore described as unclassifiable.

#### Mapped offender characteristics

Chi-square analyses were computed to determine if offender and victim characteristics could be linked to a specific dominant theme. Kruskal–Wallis test analysis was computed for continuous variables. Hybrid and Unclassifiable cases were excluded from this analysis. When mapping offender characteristics, only solved cases were used because they contained offender characteristics (*n* = 68).

No significant association was found between dominant themes and offender or victim age, ethnicity, or relationship between offender and victim. However, significant associations were found between prior conviction information and dominant themes (Table 3

**Table 3.** Dominant Theme Distribution Across Offender Characteristics.

Offender Characteristics	Involvement (*n* = 8), *n* (%)	Hostility (*n* = 10), *n* (%)	Control (*n* = 19), *n* (%)	*p*-value	*χ*^2^ value
Any preconviction	3 (38)	9 (90)	16 (84)	.017	8.200
“Theft and Kindred Offence Other”	2 (25)	1 (10)	11 (58)	.029	7.105

): χ^2^(2) = 8.200; *p* = .017. The association was moderately strong (Cohen, 1988), Cramer’s *V* = .471 and separate Chi-square analysis was conducted to explore this significance. Findings suggested “Involvement” offenders were significantly less likely to have a prior conviction, whereas individuals exhibiting either “Control” or “Hostility” themes were significantly more likely to have been convicted for a prior crime.

Chi-square analyses were then computed between type of prior conviction and dominant theme. A significant association was found between dominant themes and “theft and kindred offense other” preconviction: χ^2^(2) = 7.105; *p* = .029. This association was found to have a medium effect, Cramer’s *V* = .438. Chi-square analysis was conducted and significance was found to lie between “Hostility” and “Control”: Fishers exact test, *p* = .019. “Control” offenders were significantly more likely to have a prior conviction for “theft and kindred offence other,” while “Hostile” offenders were less likely to display a prior conviction of this type.

## Discussion

Previous literature investigating sexual offending against older adult victims has been sparse, with exploration of crime scene behaviors being even further limited. Results from this study build on previous literature regarding base rate information and have provided a thematic model from which distinct themes were identified and offender prior conviction information was mapped, successfully fulfilling the aims of the study in furthering understanding of sexual offending against the older adult.

Analysis took place in four key stages. First, frequency analysis provided base rate information confirming previous findings concerning likely prior convictions of offenders (Safarik et al., 2002) and typical location of offense (Lea et al., 2011). Here, new information was found such as the level of disrobement by offenders and victims (perhaps partially explained by victims sleeping at the time of the attack in 20% of cases), and typical injuries to victims. Results also added to current debate regarding violence and age. Within the sample, violence was typically used to gain compliance, excessive force rarely used and overkill violence never demonstrated. This finding may support research similar to Ball and Fowler (2008), who found older adults did not experience higher levels of violence when compared with other victim groups. Minimal levels of force showed the highest frequency (just under 25%) and only 3% were recorded as severe force. However, as these categories were precoded by SCAS, it is difficult to unpack the potential differences experienced by the victim, and the likely psychological impact on those particularly vulnerable. Further research is required here, especially given that national data for future survey publications will now include those over age of 60 years. The mean age of convicted offenders in the sample was 31.74 years, older than the 28.72 years average age of stranger rape offenders found by Almond et al. (2018). This study also found a larger range of ages, particularly displaying more offenders over 50 years, than those found in previous studies on general sexual offending (Barbaree et al., 1994).

Novel findings also emerged. For example, in this study, most recorded offenses were committed by strangers which contrasts with official statistics indicating only 13% of sexual offenses are committed by a stranger (ONS, 2018). However, this anomaly may be due to SCAS data collection criteria. The preponderance of stranger involvement could be interpreted as reflecting a higher risk of opportunistic attack for older adults due to their vulnerability (Salfati & Canter, 1999), whereas age of victim has been found to play less of a part in impulse offending (Goodwill & Alison, 2007). However, in this sample, a higher frequency of offenses took place in the victim’s residence potentially suggesting a degree of planning, increasing the likelihood of undisturbed access and opportunity to commit the offense. In this study, higher levels of force were more likely to occur within a residence or care home, reflecting similar findings in respect of child sexual offenses where a key factor in a heightened level of violence and abuse was the opportunity for unsupervised, prolonged access (Long et al., 2013). However, the notion of opportunistic burglars, especially given the presence of valuable and personal property stolen, should also be considered. It is difficult to disentangle which of the offenses was the dominant theme and initial intention for these offenders (burglary/theft or sexual offense). This may require further investigation alongside larger samples of pre- and postconviction data.

In the second stage of this study, analyses attempted to identify themes within offending behavior. Three themes, consistent with previous sexual offending research, were identified within the SSA; *Control*, *Involvement*, and *Hostility*. This finding supports previous arguments that older adult sexual offenders are not a homogeneous group (Burgess et al., 2007). Our model demonstrated that for some offenders *Control* behaviors are consistently used to achieve their sexual gratification (*Control*). In contrast, for other offenders, the creation of a pseudo-intimate relationship is important, and conventional sexual behaviors and high levels of interaction reflect this (*Involvement*). Other offenders seek to demonstrate significant violence through both physical and emotional aggression (*Hostility*). Unexpected findings as previously discussed were still present; the inclusion of “compliments” in the *Control* theme and “victim enjoyment” in *Hostility* were inconsistent with previous findings of sexual offending subgroups. However, the findings as a whole suggest that for the most part, the themes identified are fairly consistent with other forms of sexual offending in the literature. The presence of two behaviors, usually identified with *Involvement*, in themes of *Control* and *Hostility*, may perhaps suggest that offenders show more interaction across themes. This is a potential theory that would benefit from future research, including comparison studies between various sex offender subgroups.

The third stage of our analyses investigated the ability of the identified dominant themes to describe individual cases. Fifty-six percent of cases could be allocated to a dominant theme, supporting the model identified within the SSA output. It is perhaps notable that the proportion of perpetrators allocated a dominant theme is smaller than in previous studies (Almond et al., 2014); however, the removal of all variables under 10% rather than the usual 5% frequency may have influenced allocation. The theme of *Involvement* was allocated most frequently, displaying similarity to female offenders (Almond, McManus, Giles, & Houston, 2017). This indicates that an attempt at a consensual relationship and intimacy is important to some sexual offenders against older adult victims. The high frequency of *Involvement* cases may also reflect the victims’ vulnerability, demonstrating parallels to themes allocated to pedophiles (Canter et al., 1998).

Gerontophilia work has tended to focus on case studies (Kaul & Duffy, 1991) with an associated lack of consensus on definition, prevalence, and etiology. Thus, there is limited knowledge about how offense behaviors and offender characteristics may differ across the various identified chronophilia. Further investigation is required to understand whether the potential vulnerability of younger (pedophilia, nepiophilia, hebephilia, ephebophilia) and older victim groups (gerontophilia) in terms of decreased ability to fight back and unsupervised access (residence, care home), may result in similar characteristics and crime scene behaviors among sex offenders against these two groups of victims. Similarities include prolonged and increasingly violent attacks. In addition, causation theories may also crossover between the two groups of offenders in terms of “early sexual experiences/abuse,” and “displacement of incestuous wishes” (Ball, 2005).

Key findings emerged in the fourth and final stage of analysis when mapping dominant themes onto prior convictions. For example, offenders demonstrating an *Involvement* theme were significantly less likely to have a prior conviction, perhaps reflecting the apparent aim of *Involvement* offenders to achieve a consensual relationship (Almond, McManus, Giles, & Houston, 2017) so that these offenders did not see their actions as a criminal act. In contrast, *Control* offenders were significantly more likely than *Hostile* offenders to have a “theft and kindred offence other” prior conviction. Although an interesting finding, difficulties arise with the scale of the prior conviction title “other,” which could include around 900 potential crime types, obviously limiting research validity and practical application. However, as the data represent information provided to police, applications within law enforcement may still be useful.

### Limitations

Although the SCAS database is a valuable representative source for sampling reported sexual offenses, there is difficulty in generalizing the findings to unreported incidents. It has been established that reporting of sexual offenses remains low (ONS, 2018). This effect may be larger in older adult populations where underreporting is extremely high (Bows & Westmarland, 2015) due to factors such as the “real-rape” stereotype. Therefore, this study can only provide insight into a small section of the total number of sexual offenses committed against the older adult within the United Kingdom. It remains possible that unreported offenses may differ in their characteristics from those reported to police. For example, undetected perpetrators may demonstrate higher levels of criminal sophistication or control behaviors which prevent reporting or conviction. In addition, police data are limited in scope because the level of collected information is not as rigorous as the level of data collected in academic research. The police data may have been inconsistently gathered across varying police forces. However, despite these limitations, using the SCAS database provides data exclusively representative of the assaults reported to police. This facilitates one aim of the study of informing future criminal investigation of such offenses.

Additional sampling limitations include the small sample size. It is generally agreed that older adult victims are a minority of sexual offending victims (Bows, 2018). Thus, a smaller sample size should not be unexpected, and 143 cases was sufficient for the thematic analysis undertaken. However, sample size may have been a factor in the limited findings of links between offender/victim characteristics and dominant themes as it is difficult to state whether characteristics low in frequency are uncommon, or simply a reflection of the small sample extracted. An investigation of a larger sample would confirm and build on the present findings.

### Implications

Currently, Behavioral Investigative Advisers (BIAs) work within the National Crime Agency providing case-specific advice based on empirical evidence (Alison & Rainbow, 2011). This study furthers the knowledge of the themes employed by older aged sexual offenders and can provide BIAs and others with additional tools concerning typical characteristics of offense, offender, and the crime scene behaviors. For example, findings of this work can be applied to nominal prioritization matrixes.

Applications for treatment are also possible, findings indicate that this type of offender displays significant variance in behaviors, and a single “one-fits-all” treatment program is therefore unlikely to be sufficient in rehabilitation. Identified themes could provide a reliable understanding of crime scene behaviors across varying practitioners and can be used to tailor treatment programs specific to the dominant theme shown by the offender (Almond et al., 2014).

### Future Research

Due to limited crime scene data available from the United Kingdom for this study, it may be beneficial to collaborate with European and/or international law enforcement agencies for replication and validation with larger sample. In addition, research using a different sample base could be useful, for example, data from adult protective service agencies, rape crisis centers, or census data could provide insight into potential difference between offenses reported and not reported to police. Current theory holds that older adult victims are less likely to report offenses (Bows & Westmarland, 2015), but the nature and scale of sexual offending against older adults is not yet fully understood or represented in the literature. In addition, SCAS criteria are limited in this area as they tend to focus on “stranger” type offenses. Techniques deployed in this study, therefore, offer future research possibilities to interrogate existing theory, enhance understanding, and broaden the academic approach to this area of study.

### Conclusion

This study presents the first thematic analysis of sexual offending against the older adult based on empirically tested police data provided by SCAS. Frequency information has provided further detail to the understanding of typical offending behavior within these cases. Present findings reflecting those of previous studies of sexual offending against adult female victims in general, indicated a variance of offense behaviors present in sexual crime scenes. These behaviors can be separated into three principal distinct themes; *Control*, *Hostility*, and *Involvement*. The largest proportion of offenders showed a need for intimacy from the encounter (*Involvement*), while others seemingly displayed aggression *(Hostility*). This may reflect the increased fragility of older adult victims because prior research found the presence of broken bones (Stöckl & Penhale, 2015). Other offenders displayed a desire for controlling behavior to achieve sexual gratification (*Control*), with this cohort also having the highest number of criminal antecedents.

In this study, links were found between the three dominant themes and offender behavioral characteristics, so could provide beneficial information to law enforcement agencies. For example, *Involvement* behaviors are found at the crime scene suggests the offender is less likely to have a prior conviction. Future research, using larger, varied data sets may identify additional links between offender characteristics and crime scene behaviors. Robust data on the scale and prevalence of sexual offending against older adults are required to develop academic understanding, enhance professional investigative practice, and highlight the issue within the public domain. The findings of this study, highlighting the frequency of sexual offending against older adult victims may raise both the public and academic awareness of this type of crime. By conducting the study and suggesting further avenues of research, it is hoped that reporting may increase and future data will build a better understanding of these offenses, thus generating more effective preventive and prioritization strategies to safeguard potential victims.
